# Long-term trends of suicide by choice of method in Norway: a joinpoint regression analysis of data from 1969 to 2012

**DOI:** 10.1186/s12889-016-2919-y

**Published:** 2016-03-11

**Authors:** Quirino Puzo, Ping Qin, Lars Mehlum

**Affiliations:** National Centre for Suicide Research and Prevention, Faculty of Medicine, University of Oslo, Oslo, Norway

**Keywords:** Suicide, Methods, Changing trends, Epidemiology, Joinpoint analysis

## Abstract

**Background:**

Suicide mortality and the rates by specific methods in a population may change over time in response to concurrent changes in relevant factors in society. This study aimed to identify significant changing points in method-specific suicide mortality from 1969 to 2012 in Norway.

**Method:**

Data on suicide mortality by specific methods and by sex and age were retrieved from the Norwegian Cause-of-Death Register. Long-term trends in age-standardized rates of suicide mortality were analyzed by using joinpoint regression analysis.

**Results:**

The most frequently used suicide method in the total population was hanging, followed by poisoning and firearms. Men chose suicide by firearms more often than women, whereas poisoning and drowning were more frequently used by women. The joinpoint analysis revealed that the overall trend of suicide mortality significantly changed twice along the period of 1969 to 2012 for both sexes. The male age-standardized suicide rate increased by 3.1 % per year until 1989, and decreased by 1.2 % per year between 1994 and 2012. Among females the long-term suicide rate increased by 4.0 % per year until 1988, decreased by 5.5 % through 1995, and then stabilized. Both sexes experienced an upward trend for suicide by hanging during the 44-year observation period, with a particularly significant increase in 15–24 year old males. The most distinct change among men was seen for firearms after 1988 with a significant decrease through 2012 of around 5 % per year. For women, significant reductions since 1985–88 were observed for suicide by drowning and poisoning.

**Conclusions:**

The present study demonstrates different time trends for different suicide methods with significant reductions in suicide by firearms, drowning and poisoning after the peak in the suicide rate in the late 1980s. Suicide by means of hanging continuously increased, but did not fully compensate for the reduced use of other methods. This lends some support for the effectiveness of method-specific suicide preventive measures, such as restrictions to the access to firearms, which had been implemented in Norway during the relevant time period.

## Background

According to data from the World Health Organization, over 800 000 people died from suicide worldwide in 2012, representing a global age-standardized suicide rate of 11.4 per 100 000 population [1]. Common methods of suicide include pesticide poisoning, which accounts for around 30 % of the global suicides and occurs mostly in rural agricultural areas in low- and middle-income countries, as well as hanging and firearms [2]. A number of countries have observed significant changes in the distribution of suicides by method [3–8], mostly due to changes in availability and sociocultural acceptability of different suicide methods [9]. Accurate knowledge of the most commonly used suicide methods across time is important as a basis for developing and selecting prevention strategies and for contributing to the understanding of the epidemiology of suicide.

In Norway, a country where an average of 530 suicides are registered every year [10], some changes in the patterns of suicide methods used over time have been reported. Mehlum et al. [11] reported a remarkable change from intoxication to the use of hanging/strangulation in adolescents and young adults in the period 1973–1994. A recent study by Gjertsen et al. [12] analyzed the trends of suicide by firearms among males between 1969 and 2009 and suggested that the significant decrease in male firearm suicides in Norway after 1990 might be due to stricter firearms legislation. Despite that some reports on changes in certain specific suicide methods have been published a systematic analysis of trends in suicide mortality with special reference to the victim’s choice of method in Norway has so far not been published.

The joinpoint regression model is useful in order to identify and describe the occurrence of changes in distinct periods of time in trend data [13]. In the present study we wanted to use this model to identify significant changing points of method-specific suicide mortality in Norway from 1969 through 2012 in order to enhance our understanding of long-term trends of suicide as a basis for strategies and programs for suicide prevention.

## Methods

### Data sources and study population

Data on suicide deaths, specific methods used for suicide and sex and age of the deceased subjects were retrieved from the Norwegian Cause-of-Death Register for the years from 1969 through 2012. We selected this time span because personal data in this register became computerized and thus accessible for research from the year 1969. Suicide rates were further calculated by sex and for eight age groups: 15–24, 25–34, 35–44, 45–54, 55–64, 65–74, 75–84 and 85 years and older. In Norway, suicides were coded according to the International Classification of Diseases (ICD), 8th Revision for the years 1969–1985 (codes E950-E959), 9th Revision for the years 1986–1995 (codes E950-E959) and 10th Revision for the years 1996–2012 (codes X60-X84, Y870) [14]. The different suicide methods were classified into seven categories: “Poisoning” (E950-E952 or X60-X69), “Hanging” (E953 or X70), “Drowning” (E954 or X71), “Firearms and explosive material” (E955 or X72-X75), “Cutting or piercing instruments” (E956 or X78), “Jumping from a high place” (E957 or X80) and “Other or unknown method” (E958-E959 or X76-77, X79, X81-84, Y870). The category “Firearms and explosive material” was relabeled as “Firearms” in the text since suicides in this group almost exclusively contained suicides by firearms in Norway. Data on the yearly national population for the entire observation period were derived from the statistics published on the website of Statistics Norway [15].

### Statistical analyses

Overall significant differences in methods for suicide by sex and age groups were assessed using Chi-square test for association. We calculated age-standardized rates (ASRs) by the direct method of standardization using the age distribution of the 2011 Norwegian census population as the standard since this census population was the most recent one available in Norway and since it had quite similar age distributions to the previous 2001 census population [16]. ASRs were expressed as number per 100 000 population.

Long-term trends in ASRs of suicide mortality in Norway were analyzed using joinpoint regression analysis [13]. This method describes changes in data trends by connecting several different line segments on a log scale at “joinpoints”, and can identify points where a statistically significant change over time in the linear slope of the trend occurred. The analysis starts with the minimum number of joinpoints (i.e., 0 joinpoint, which is a straight line) and tests whether one or more joinpoints are statistically significant and must be added to the model. The tests of significance use a Monte Carlo permutation method. In addition, an annual percentage change (APC) in ASRs for each line segment was estimated. The APC is tested to determine if it is different from the null hypothesis that the annual percent change is 0 %. In the final model, each joinpoint indicates a statistically significant change in trends (increase or decrease) and each of those trends is described by an APC. The average annual percentage change (AAPC) and the related 95 % confidence interval (95 % CI) were also computed for the full study period 1969–2012 in order to have a summary measure of the trend over the complete period of study. The AAPC is a weighted average of the APCs, with the weights equal to the length of the joinpoint segments. The APC and AAPC were calculated separately for men and women and stratified for suicide method by all subjects and by age groups (15–24, 25–44, 45–64 and ≥65). For all analyses, a *P* value less than 0.05 was considered to be statistically significant.

The computation of suicide mortality rates and statistical analyses were performed in R software (Version 3.2.1). Joinpoint analyses were performed using the Joinpoint software (Version 4.2.0.2) from the Surveillance Research Program of the US National Cancer Institute [17].

### Ethical considerations

Access to data for the study was approved by the Regional Ethical Committee for Medical and Health Research (REK sør-øst) and the Norwegian Institute of Public Health (FHI).

## Results

### General description

During the 44-year observation period a total of 22 914 suicides for subjects aged > = 15 were recorded. Among them, 16 728 were male (73 %) and 6 186 were female (27 %). The most common suicide method during the entire observation period was hanging, followed by poisoning and firearms (Table 1). In total, these methods were used in 80 % of all suicides in Norway. Men chose suicide by firearms more often than women, whereas poisoning and drowning were more frequently used by women. Overall, there was a significant sex difference in methods used for suicide (Chi-square = 3042.5, *P* < 0.001).

**Table 1:** Distribution of suicides by method among men and women in Norway, 1969–2012

	Males		Females		Both sexes	
Method	n	%	n	%	n	%
Hanging	5 534	33.1	1 630	26.3	7 164	31.3
Poisoning	3 345	20.0	2 501	40.4	5 846	25.5
Firearms	5 073	30.3	169	2.7	5 242	22.9
Drowning	922	5.5	1 026	16.6	1 948	8.5
Jumping from a high place	658	3.9	421	6.8	1 079	4.7
Cutting or piercing instruments	450	2.7	141	2.3	591	2.6
Other or unknown method	746	4.5	298	4.8	1 044	4.6
*total*	16 728	100	6 186	100	22 914	100

**Fig. 1:**
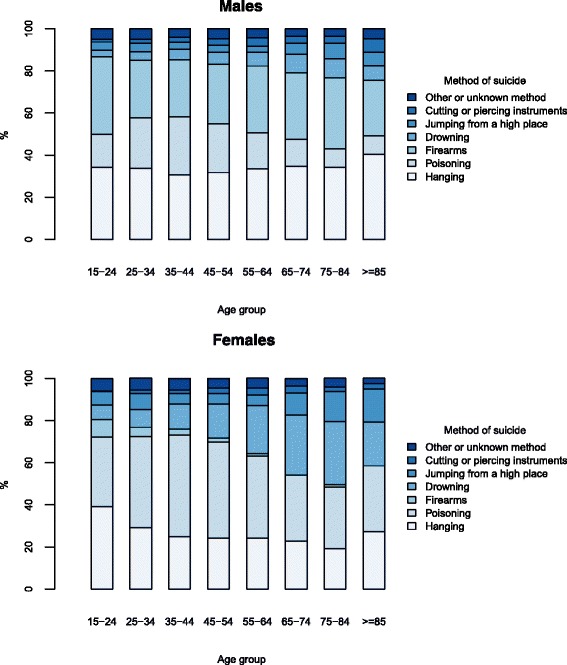
Distribution of suicides by method in each age group for men and women, Norway, 1969–2012

Figure 1 depicts the distribution of suicide methods in each age group. Visual inspection shows that the rectangles in each age group are not homogeneously distributed, indicating dependency between suicide methods and age groups (Chi-square = 577.8, *P* < 0.001 for males and Chi-square = 563.8, *P* < 0.001 for females). Among persons aged 15–24 years the most common method of suicide was firearms for males (37 %) and hanging for females (39 %). Among female suicides, more than 40 % of victims in the age groups 25–34, 35–44 and 45–54 died by poisoning, whereas 30 % of those aged 75–84 used drowning to take their own life. For male suicides, more than 30 % of the victims in each age group died by hanging and around 28 % of those aged 35–44 used poisoning to commit suicide.

### Trends of overall suicide mortality

**Fig. 2:**
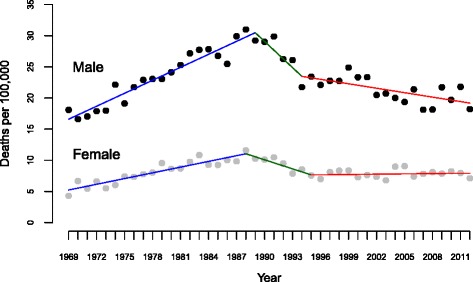
Trends in age-standardized suicide rates from 1969 to 2012 in men and women in Norway (data markers represent observed rates; lines have been drawn according to the joinpoint results)

The age-standardized suicide rates (per 100 000) in males and females for all suicide methods are presented in Table 2 and Fig. 2. The average of annual age-standardized suicide rates for the period of study 1969–2012 was almost 2.8 times higher among males than that among females (22.9 per 100 000 in males vs 8.2 per 100 000 in females). Our joinpoint analysis showed that the male ASR increased by 3.1 % per year until 1989 and decreased by 1.2 % per year between 1994 and 2012. In the period 1989–1994, the decrease in suicide rate shown in Fig. 2, even though striking, was not statistically significant (*P* = .055). Among females, the long-term suicide rate increased by 4.0 % per year until 1988, decreased by 5.5 % through 1995, and then stabilized.

**Table 2:** Estimates from joinpoint analyses of age-standardized suicide rates for men and women in Norway, 1969–2012

		Joinpoint analyses^a^: 1969–2012							
	Average Annual ASR (1969–2012)	Trend 1		Trend 2		Trend 3		Full range	
		Years	APC	Years	APC	Years	APC	AAPC (95 % CI)	*P* value
*Male*	22.9	1969–1989	3.1*	1989–1994	−5.6	1994–2012	−1.2*	0.3 (−0.5 to 1.0)	0.481
*Female*	8.2	1969–1988	4.0*	1988–1995	−5.5*	1995–2012	0.1	0.9 (−0.0 to 1.8)	0.057

Abbreviation: *ASR* age-standardized rate, *APC* annual percentage change, *AAPC* average annual percentage change

^**a**^Joinpoint Regression Program version 4.2.0.1 (National Cancer Institute/US National Institutes of Health, Bethesda, Md). The annual percentage change and the average annual percentage change are based on rates that were age-standardized to the 2011 Norwegian census population

*The annual % change (APC) is statistically significantly different from zero (*P* < .05)

We also performed the joinpoint analysis only focusing on the last two decades (1994–2012), but no differences in trends were observed during this period compared to the analysis over the complete period of study.

**Fig. 3:**
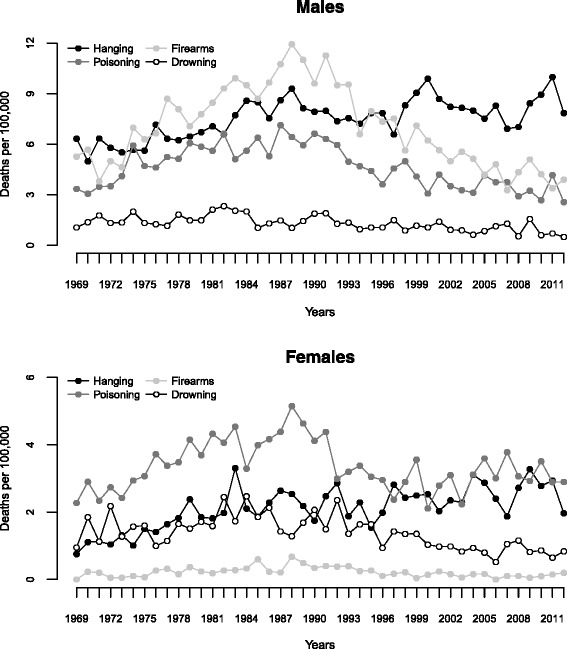
Age-standardized suicide rates for the four most common methods for suicide in Norway, 1969–2012

Figure 3 displays how the age-standardized suicide rate by the four leading suicide methods changed over the entire time period. The results of joinpoint analyses on ASRs for suicidal methods are presented in Table 3.

**Table 3:** Estimates from joinpoint analyses of age-standardized suicide rates by specific suicide methods in Norway, 1969–2012

		Joinpoint analyses^a^: 1969–2012							
		Trend 1		Trend 2		Trend 3		Full range	
	Age group	Years	APC	Years	APC	Years	APC	AAPC (95 % CI)	*P* value
*Male*									
Hanging	15–24	1969–1971	99.4*^b^	1971–1999	5.5*	1999–2012	−0.5	6.8 (3.5 to 10.1)	0.000
	25–44	1969–2012	2.5*					2.5 (2.0 to 2.9)	0.000
	45–64	1969–1988	2.0*	1988–1993	−9.3	1993–2012	0.5	−0.0 (−1.7 to 1.6)	0.961
	≥65	1969–1993	0.7	1993–2012	−3.9*			−1.3 (−2.6 to −0.1)	0.035
	*total*	1969–1985	2.4*	1985–2012	0.2			1.0 (0.5 to 1.5)	0.000
Poisoning	15–24	1969–1979	10.3*	1979–2005	−1.6	2005–2012	−18.5*	−2.0 (−4.4 to −0.4)	0.106
	25–44	1969–1979	8.9*	1979–2012	−2.5*			0.0 (−1.2 to 1.3)	0.971
	45–64	1969–1983	5.3*	1985–2012	−3.2*			−0.5 (−1.6 to 0.7)	0.399
	≥65	1969–1997	2.8*	1997–2000	−30.2^b^	2000–2012	9.1*	1.7 (−6.6 to 10.8)	0.690
	*total*	1969–1982	5.7*	1982–2012	−2.7*			−0.3 (−1.0 to 0.5)	0.507
Firearms	15–24	1969–1988	10.8*	1988–2012	−9.3*			−0.9 (−2.2 to 0.4)	0.176
	25–44^c^	1969–1971	−20.4^b^	1971–1988	4.8*	1988–2012	−5.8*	−2.5 (−5.0 to 0.1)	0.064
	45–64	1969–1987	3.3*	1987–2012	−4.3*			−1.2(−2.2 to −0.2)	0.017
	≥65	1969–1987	6.3*	1987–2012	−1.6			1.6 (0.1 to 3.0)	0.029
	*total*	1969–1988	4.6*	1988–2012	−4.8*			−0.8 (−1.4 to −0.1)	0.016
Drowning	*total*	1969–1982	2.6	1982–2012	−3.0*			−1.3 (−2.7 to 0.0)	0.058
Jumping from a high place	*total*	1969–2012	0.8*					0.8 (0.2 to 1.4)	0.007
Cutting or piercing instruments	*total*	1969–2012	−0.6					−0.6 (−1.2 to 0.1)	0.078
Other or unknown method	*total*	1969–2012	4.8*					4.7 (3.6 to 6.0)	0.000
*Female*									
Hanging	*total*	1969–1979	9.3*	1980–2012	0.7*			2.7 (1.6 to 3.7)	0.000
Poisoning	*total*	1969–1988	3.5*	1988–1997	−6.3*	1997–2012	1.3	0.6 (−0.5 to 1.7)	0.272
Firearms^d^									
Drowning	*total*	1969–1985	3.0*	1985–2012	−3.7*			−1.3 (−2.4 to −0.1)	0.033
Jumping from a high place	*total*	1969–1988	4.2*	1988–2012	−2.2*			0.6 (−1.2 to 2.4)	0.519
Cutting or piercing instruments^d^									
Other or unknown method^d^									

Abbreviation: *APC* annual percentage change, *AAPC* average annual percentage change

^a^Joinpoint Regression Program version 4.2.0.1 (National Cancer Institute/US National Institutes of Health, Bethesda, Md). The annual percentage change and the average annual percentage change are based on rates that were age-standardized to the 2011 Norwegian census population

^b^The result is not reliable due to few observations between joinpoints

^c^The model with two joinpoints was preferred to the one with four joinpoints because of model fitting

^d^Joinpoint cannot process records with dependent variable = 0

*The annual % change (APC) is statistically significantly different from zero (*P* < .05)

### Changes in method-specific suicide in males

Over the 44-year observation period, male suicide by hanging increased significantly with an average annual percentage change of 1.0 % (95 % CI: 0.5 to 1.5). This increase in suicide by hanging was especially strong in 15–24 year old males with an AAPC of 6.8 % (95 % CI: 3.5 to 10.1). Within this age group, a slightly larger increase (data not shown) was observed in 20–24 year old males (AAPC = 8.2 %, 95 % CI: 3.5 to 13.2) compared to subjects aged 15–19 years (AAPC = 6.8 %, 95 % CI: 2.8 to 11).

Suicide rates by poisoning followed an increasing trend from 1969 to 1982 (APC = 5.7 %) and then decreased continuously through 2012 with an APC of −2.7 %.

Firearm suicide rates in males were nonlinear, with an inverse “U”-shaped trend that reached a maximum in 1988; the ASRs first increased with an annual change of 4.6 % during the period 1969–1988 and then decreased through 2012 with approximately the same APC (= −4.8 %). The largest increase in the number of suicides by firearms between 1969 and 1988 was observed in the 15–24 age group.

Suicide by drowning was stable from 1969 to 1982 and then followed a decreasing trend through 2012 (APC = −3.0 %).

### Changes in method-specific suicide in females

As shown in Fig. 3, the rate of suicide by drowning among females first increased during 1969–1985 and then decreased through 2012, showing an average annual percentage change of −1.3 % (95 % CI: −2.4 to −0.1). The rate of poisoning also followed an increase-then-decrease trend with a maximum level in 1988; however after the year 1997 it stabilized through 2012.

In contrast to the rate of suicide by poisoning and drowning, the female rate of suicide by hanging significantly increased during the entire period with an AAPC of 2.7 % (95 % CI: 1.6 to 3.7).

## Discussion

To the best of our knowledge, this is the first study quantifying suicide mortality trends by using a joinpoint regression analysis of all suicides by various specific methods in Norway from the year 1969 through 2012. Our analyses indicated that the overall trend of suicide mortality significantly changed twice along the period 1969–2012 for both sexes and showed a decline of suicide mortality after the peak prevalence years 1988–89 in Norway. Indeed, in response to the increase in suicides from the end of the 1960s, in 1989 Norwegian authorities requested the preparation of the first national plan for suicide prevention [18]. Our analyses, furthermore, revealed significant changes over the observed time periods in the use of specific methods of suicide.

Epidemiological studies have shown that the trends in choice of particular suicide method change within and between countries over time [3–8] and that these changes are unlikely to be random. People change suicide methods, perhaps in response to the changing availability of these methods in their environment [9] or their changing knowledge of the existence of such methods. Generally, whereas the most common suicide method in Europe is hanging [19], in Asia the most frequently used suicide method is self-poisoning with pesticides [20], and in the United States it is firearms, consistently accounting for nearly 60 % of all suicide deaths [21].

### Changes in suicide by hanging

In Norway, as our study revealed, hanging was the most frequently used suicide method in the total population and this method accounted for more than 30 % of all suicides. In male suicides, hanging has increased with an average annual percent change of 1.0 % over the 44-year observation period, showing a particularly strong increase of 6.8 % per year in 15–24 year olds. In female suicides, the choice of hanging as suicide method changed over the entire period with an increase of almost 3 % per year. Given the decreasing use of other suicide methods such as firearms among males and poisoning and drowning among females (Fig. 3), these findings might perhaps best be explained through the so-called substitution hypothesis, suggesting that hanging may have been a substitution method for firearms among males and for poisoning and drowning among females. It is, however, important to note that if such a substitution effect was present, it did not lead to complete substitution since the reduction in methods such as firearms and poisoning was associated with a reduction in total suicide rates.

### Changes in suicide by firearms

Female suicide by firearms was very unusual during the observation period (accounting for only 2.7 % of all female suicide deaths). We found, however, a strong decrease in the male firearm suicide rate both in the total population and in the 15–24 year olds after this type of suicide peaked in 1988. This development may be associated with a stricter firearm legislation and other preventive measures aimed at reducing suicide by firearms having been implemented in Norway in the relevant time period, as also suggested in a previous report by Gjertsen et al. [12]. During the 1990s laws were passed in Norway that made acquisition of guns more difficult and storage of firearms in insurance approved gun-safes mandatory. The decrease in firearm related suicides following firearm legislation reforms has also been shown in other countries, among them Australia [22], the Unites States [23, 24] and Canada [25]. Most researchers agree that there is evidence for the effectiveness of restricting access to means of suicide, hence reducing the method-specific, as well as the overall, suicide rate [26], at least with regard to toxic domestic gas [27, 28], firearms [23, 29], drugs [30, 31] and bridges as jumping sites [32, 33]. Therefore, reduction of the availability and lethality of methods for suicide have been proposed as important approaches to suicide prevention. A shortcoming of these approaches is however that suicidal individuals, once deprived of their preferred means of suicide, could shift to using alternative methods. A study by Caron et al. [34] analyzed the impact of new legislation implemented in Quebec (Canada) making safe storage of firearms mandatory. The study did not find evidence that the new bill improved the downward trend in suicide by firearms, which had already begun before the implementation of the law, but it did find support for the substitution hypothesis; firearm suicide was replaced by hanging among males. In our study, the evidence of a substitution effect between methods of suicide for men (change from firearms to the use of hanging) was not so clear since the decrease in firearms suicide contributed to the statistically significant decrease in the total suicide rates after the peak year 1988. The same effect was also seen among the young. This suggests that limiting access to firearms is a valuable measure to prevent suicide since, in the context of the present study, it seemed not to lead to a complete shift towards the use of other suicide methods.

### Changes in suicide by poisoning

Notable trend changes were seen for poisoning among women after 1988 with a significant decrease through 1997 of more than 6 % per year followed by a leveling off (1997–2012). This method-specific trend appeared to reflect the same changes in the female suicide rate for all suicide methods over the entire observation period. Based on these results and considering the trend of female suicide by hanging, there was no clear evidence for any substitution effect from poisoning to hanging after 1988. A remarkable change from intoxication to the use of hanging/strangulation in adolescents and young adults in Norway in the period 1973–1994 was reported by Mehlum et al. [11]. The decline in the poisoning suicide rate as well as for all suicide methods after 1988 might be associated with an increase in antidepressant sales, as also suggested by other authors. Bramness et al. [35] concluded that the reduction in suicide rates in Norway and its counties was related to the increased sales of new antidepressants. Reseland et al. [36] have however argued that even though the fall in suicide rates in Norway partly coincided with the period when newer antidepressants were introduced and their sales increased markedly, the period when the greatest increases in antidepressant sales occurred was characterized by relatively stable suicide rates. Currently, findings from empirical studies on the association between changes in the sales of newer antidepressants and changes in the suicide rates internationally are inconsistent [37–39].

### Changes in suicide by drowning

Another finding of this study is the fact that the rate of suicide by drowning among females increased during the first 16 years of the observation period and then decreased through 2012. This may suggest a substitution effect after 1995–96 between drowning and hanging. The decrease in suicide by drowning did not lead to a significant decrease in the total female suicide rate after 1995–96.

### Limitations and strengths

One limitation of the present study is that we have applied a model that fits linear segments to possibly non-linear data. Although the positions of the joinpoints of these segments provide useful estimates of the years in which trends in suicide rates changed significantly, they represent a simplification of the observed temporal trends, and should therefore be interpreted with caution. Secondly, the study was descriptive in design, and the delineation of the complex relationships among risk factors of suicide was beyond the scope of this study.

Among the strengths of the study is the long time span (1969–2012) covered and hence the possibility to provide a systematic analysis of long time trends in the choice of suicide methods in Norway. The use of the joinpoint analysis allowed statistical testing of directions and sizes of trends in suicide mortality data, detecting important changes. Indeed, compared to other regression methods used to investigate trends over time and to find the best-fit line through several years of data, the joinpoint analysis tests whether a multi-segmented line is a significantly better fit than a straight or less-segmented line. Accordingly, joinpoint analysis provides a much clearer picture of what is happening during a distinct period in specific terms (identifying the calendar years in which significant changes in trends occurred, and the annual percentage of change within the periods identified) than a single summary trend statistic or simple analysis of graphs and percentages.

## Conclusions

In conclusion, a reduction in suicides by firearms, drowning and poisoning and an increasing trend in suicide by hanging were found among both males and females in Norway. Therefore, suicide by hanging emerges as an important public health concern in both sexes. The rise in the number of deaths by hanging is not so readily explained. The increase could not be related to an increased availability of the method since the means have always been easily accessible to everyone, with the exception of certain hospitalized or incarcerated individuals. The only plausible explanation to the increased use of this method seems to be that other methods have become less readily available. Despite the relative success of preventing suicide by restricting access to firearms, the method-specific approach will have limited effect on preventing suicide by hanging in the general population of non-hospitalized and non-incarcerated individuals since the methods needed for this type of suicide are found everywhere in people’s daily environment. Prevention of suicide by hanging must in addition rely, as all other suicide prevention [26], on strategies for more effective identification of at-risk individuals and engaging these individuals and families in treatments and interventions that are cost-effective and available, and on strategies to reduce incidence rates of mental ill-health and other important factors associated with an increased suicide risk in the population.

## Abbreviations

95 % CI: 95 % confidence interval
AAPC: average annual percent change
APC: annual percent change
ASRs: age-standardized rates
